# Comparative analysis of primary metabolites and transcriptome changes between ungrafted and pumpkin-grafted watermelon during fruit development

**DOI:** 10.7717/peerj.8259

**Published:** 2020-01-06

**Authors:** Ali Aslam, Shengjie Zhao, Muhammad Azam, Xuqiang Lu, Nan He, Bingbing Li, Junling Dou, Hongju Zhu, Wenge Liu

**Affiliations:** 1 Zhengzhou Fruit Research Institute, Chinese Academy of Agricultural Sciences, Zhengzhou, Henan, China; 2 Institute of Horticultural Sciences, University of Agriculture, Faisalabad, Punjab, Pakistan

**Keywords:** *Citrullus lanatus*, Grafting, qRT-PCR, Metabolism, Transcriptome, Organic acids, Sugar, Amino acid

## Abstract

Grafting has been reported as a factor that influences fruit quality. However, a comprehensive study of the metabolic profile related to fruit quality and the underlying molecular mechanism in grafted watermelon has not been carried out. Metabolomics and transcriptome analysis were performed on both pumpkin-grafted watermelon and ungrafted watermelon at different developmental stages. In total, 56 primary metabolites were identified with either high or low abundance between ungrafted and pumpkin-grafted watermelon. The results indicated that ornithine, arginine, lysine (amino acids), glucose, sucrose, glucosamine (sugars), malic acid, fumaric acid and succinic acid (organic acids) were among the dominant metabolites influencing fruit quality. Additionally, comparative RNA sequence analysis on grafted and ungrafted watermelon yielded 729, 174, 128 and 356 differentially expressed genes at 10, 18, 26 and 34 days after pollination (DAP), respectively. Functional annotations of these genes indicated that grafting significantly altered the biological and metabolic processes related to fruit quality. Our comparative metabolomics and transcriptome analysis revealed that *FBA2, FK, SuSy, SPS, IAI, AI* and sugar transporter gene (*SWT3b*) might play a central role in the accumulation of glucose and sucrose, whereas higher malic acid content was attributed to high down regulation of *ALMT13* and *ALMT8* in pumpkin-grafted watermelon. Changes in the ornithine, glutamine, alanine, tyrosine, valine, asparagine, phenylalanine, arginine and tryptophan contents were consistent with the transcript level of their metabolic genes such as *NAOD, GS, AGT, TaT, aDH1*, *OGDH, aDC, 4CL 1, PaL, CaT* and two nitrate transporter genes (*NRT1*) in pumpkin-grafted watermelon. This study provides the basis for understanding the graft-responsive changes in the metabolic profile and regulatory mechanism related to fruit quality.

## Introduction

Watermelon (*Citrullus lanatus* L.*)* is an important horticultural crop and its production accounts for approximately 9.5% of total vegetable production worldwide. Nearly 83.7% of watermelon production is in Asia (FAOSTAT, 2017). China is the leading producer of watermelon with more than half of the world’s production, and around 20% of crops come from grafted plants (Davis et al., 2008). Watermelon fruit provides an enormous amount of health-promoting nutrients such as vitamins, minerals, fiber, antioxidants, carotenoids, citrulline, and flavonoids (Collins et al., 2007; Hayashi et al., 2005; Perkins-Veazie et al., 2006). Many factors such as fruit size, shape, rind color, thickness, flesh color, texture, sugars, aroma, flavor and minerals play important roles in regulating the quality of watermelon. Sugar, organic acids and amino acids are among the key metabolites that determine fruit quality (Kader, 2008). Glucose, fructose and sucrose are predominant sugars, and sucrose accumulates as a consequence of a reduction in glucose and fructose, and accounts for 70% of total reducing sugars in mature watermelon fruit (Guo et al., 2011; Yativ, Harary & Wolf, 2010). Citric acid, malic acid and oxalic acid are regarded as major organic acids in watermelon (Gao et al., 2018). In addition, amino acids constitute a large portion of watermelon nutritional profile, and each amino acid is recognized for its specific taste. These components are also involved in both primary metabolism and biosynthesis of secondary metabolites, including vitamins and aroma volatiles which further influence fruit quality (Wang et al., 2008; Zhang & Ruan, 2016).

Grafting is an innovative and friendly technique commonly used in continuous cropping systems around the world. Grafting has gained tremendous importance in the global horticulture industry, and demand of grafted plants has increased dramatically due to high yield and increased resistance against biotic and abiotic stresses (Rouphael et al., 2017). The key objective of grafting is to increase yield and production (Condurso et al., 2012; Davis & Perkins-Veazie, 2005; Davis et al., 2008), and also to fulfill the needs of the growing global population (Liu et al., 2013). Moreover, grafting has been used to inhibit the effects of soil pathogens (Lee, 1994), soil-borne diseases (Rouphael et al., 2017), to increase vegetable nutrient uptake, to reduce the negative effects of mineral (boron, copper, cadmium), toxicity (Colla et al., 2010; Rouphael et al., 2008; Savvas et al., 2009; Venema et al., 2008), and to enhance fruit quality (Aloni et al., 2010). Grieneisen et al. (2018) reported that grafting in tomato improve the fruit quality (firmness, lycopene, soluble solid contents, vitamin C, pH and taste) and 33% of commercial rootstocks showed promising result against soil-borne pathogens.

Grafting can influence endogenous production of primary and secondary metabolites such as sugars, acids, volatiles and vitamins, which in turn regulate the fruit quality. For example, grafting in muskmelon caused a reduction in total amino acid content in mature fruit, thus affecting the fruit quality (Liu et al., 2010). Grafted watermelon showed higher malic acid content, while citric acid content decreased along the developmental stages (Fredes et al., 2017). Grapes accumulated significantly higher contents of several amino acids such as isoleucine, serine, valine, glutamine, leucine, arginine, and threonine following grafting (Lee & Steenwerth, 2011). The previous study indicated that grafting of “Synda” tomato plants onto “King Kong” rootstock increased vitamin C and total soluble solids (TSS) contents (Rahmatian, Delshad & Salehi, 2014). In citrus, effect of rootstock exposed significant differences in the concentration of some primary and secondary metabolites. The level of polyphenolics and limonoidaglycones increased by using sour orange rootstock. While “Rough lemon” moderately increased the vitamins (B-complex and C), aroma volatiles and polyphenolics (Saini et al., 2019). In cherries, CAB 11E rootstock influence fruit sugar composition, higher levels of anthocyanins and polyphenols were recorded in the grafted scion (Spinardi, Visai & Bertazza, 2001). SSC and titratable acidity were found to be higher in “Boludo” scion grafted onto wild tomato *(Solanum cheesmaniae*) and the cultivated tomato rootstocks. While “Radja” rootstock increased the fruit yield and quality traits of “Boludo” scion (Flores et al., 2010).

Similarly, the positive or negative impacts of grafting on morphological properties, soluble sugars, organic acids and TSS have been reported in various crops (Bletsos & Passam, 2010). For this purpose, several cucurbit rootstocks such as *Benincasa hispida, Cucurbita maxima, Luffa cylindrica, C. ficifolia, Lagenaria siceraria, C. argyrosperma, and C. moschata* are used in watermelon production (Fredes et al., 2017; Rouphael et al., 2012, 2010). Watermelon fruit quality is largely influenced by grafting (Rouphael et al., 2010). Grafting in watermelon has been shown to influence metabolite content such as organic acids, lycopene, carotenoids and ascorbic acid. Alterations in gene expression or direct changes in metabolite translocation may underlie these grafting-induced alterations in metabolite profiles (Petropoulos et al., 2014; Proietti et al., 2008). Watermelon grafted onto squash and bottle gourd rootstock induced transcriptional changes of genes related to hormone signaling, transporters, primary and secondary metabolism, transcription factors and response to stimuli, indicating the important role of these genes in mediating biochemical events in grafted seedlings (Liu et al., 2016). In grafted cucumber, several differentially expressed genes (DEGs) were annotated to glycolysis, fructose and linolenic acid pathways, indicating that grafting significantly influenced the fruit quality in cucumber (Zhao et al., 2018). In tea, metabolomic and transcriptomic analyses revealed the up-regulation of several genes involved in flavonoid pathway, and down-regulation of theanine and caffeine metabolic genes following the grafting onto *C. sinensis* and *C. oleifera* rootstock (Deng et al., 2018). Cucumber fruit quality was significantly improved with different rootstocks and differences in quality related metabolite were regulated by the prominent DEGs involved in some primary and secondary metabolism (Miao et al., 2019).

Despite the importance of grafting in altering the metabolic processes related to fruit quality, few attempts have been made to dissect the mechanisms that underlie grafting-induced phenotypic changes. The molecular mechanism related to fruit quality affected by grafting is unclear and it remains largely unknown in watermelon. Elucidation of the mechanism influencing the fruit quality of grafted plant becomes essential because of higher consumer demand for quality.

In addition, comprehensive metabolite profiling of sugars, organic acids and amino acids is lacking during fruit development in grafted and ungrafted watermelon. Therefore, we performed comparative analysis of primary metabolites and transcriptome changes between ungrafted (control) and pumpkin-grafted watermelon during fruit development in order to gain insight into the role of grafting in molecular processes. Our findings may provide the basis for further investigation of the physiological function of identified candidate genes in the mediation of fruit quality in grafted watermelon.

## Materials and Methods

The plants were grown in a plastic greenhouse of size 85 × 8 × 3 meters from March to July (2018) in Zhengzhou Fruit Research Station in Xin Xiang County, Henan, China. This field is under watermelon cultivation from last four years. Watermelon (*C. lanatus* (Thunb.) Matsum & Nakai var. *lanatus)* diploid mini watermelon F1 hybrid cultivar “Zhongyu No. 1” characterized by a green skin with dark green stripes and yellow flesh was grafted onto F1 hybrid pumpkin “Xi Jia Qiang Sheng” (*Cucurbita moschata*). Ungrafted watermelon served as a control. This scion variety is easy to set fruit, different from red flesh, and is a new variety. All plant materials were obtained from the Laboratory of Polyploidy Watermelon Breeding, Zhengzhou Fruit Research Institute, Chinese Academy of Agricultural Sciences. Seeds were sown in plastic trays containing peat moss, and “top insertion grafting” was carried out according to a previously reported method (Lee, 1994). Plants were shifted to a plastic greenhouse on the appearance of the third true leaf. Rows were 150 cm apart, and plant-to-plant distance was maintained at 50 cm. Randomized complete block design with three replications was used. Each plot consisted of 40 plants in a single row. Plants were trained to a single stem by clipping off side branches and were supported with rope. Only one fruit was allowed to develop on each plant.

During the growing season, standard field management procedures such as pest and disease control, weeding, fertilizer application and irrigation were implemented. On the onset of flowering, the female flowers were manually pollinated on the same day, and tagging was done to record the number of days after pollination (DAP).

According to previous studies, cultivated watermelon ripens in four critical stages, namely 10, 18, 26 and 34 DAP (Gao et al., 2018). Three uniform watermelon fruits from three independent plants at each developmental stage in each treatment were harvested (Fig. S1).

Harvested fruits were cut longitudinally into two halves, and in total, 24 fruit flesh samples were collected from the heart area (center) of the watermelon, then promptly frozen in liquid nitrogen and stored at −80 °C for future use.

In each sample, one set of flesh tissue was used for RNA extraction and the other set of flesh tissue was used for determination of sugars, organic acids and amino acids. SSC and pH (acidity) of the fruits were measured from the center of the flesh using electronic test instruments (HC-112ATC, Shanghai LICHENKEYI, China and PHB-4, Shanghai LICHENKEYI, China, respectively).

### Determination of sugars, organic acids and amino acids

#### Chemicals

In this study, all solvents such as methanol and acetonitrile (Merck, Darmstadt, Germany) were of HPLC-grade. For this study, water was double deionized with a Milli QULTRA purification system (Millipore, Vimodrone, Italy). All original standards were acquired from Sigma–Aldrich, USA (www.sigmaaldrich.com/united-states.html). Methanol and deionized water were used as the respective solvents for preparing stock solutions of organic acids, sugars and amino acids and were kept at −20 for downstream analysis.

#### Sample preparation and extraction

The freeze-dried flesh samples were crushed using a mixer mill (MM 400, Retsch) with a zirconia bead for 1.5. min at 30 Hz. A total of 100 mg powder was weighted and extracted overnight at 4 °C with one ml 70% aqueous methanol. Following centrifugation at 10,000 g for 10 min, the extracts were absorbed (CNWBOND Carbon-GCB SPE Cartridge, 250 mg, three ml; ANPEL, Shanghai, China, www.anpel.com.cn/cnw) and filtrated (SCAA-104, 0.22 μm pore size; ANPEL, Shanghai, China, http://www.anpel.com.cn/) before LC-MS analysis.

#### Analytical condition of LC-MS/MS

Analyses of sample extracts were carried out using an LC-ESI-MS/MS system (HPLC, Shim-pack UFLC SHIMADZU CBM30A system, www.shimadzu.com.cn/; MS, Applied Biosystems 6500 Q TRAP (www.appliedbiosystems.com) at Metware (Wuhan, China).

The LC-MS/MS system operated under the following conditions: HPLC: column, Waters ACQUITY UPLC HSS T3 C18 (1.8 μm, 2.1 mm × 100 mm); solvent system, water (0.04% acetic acid): acetonitrile (0.04% acetic acid); gradient program, 95:5 V/V at 0 min, 5:95 V/V at 11.0 min, 95:5 V/V at 12.0 min, 95:5 V/V at 12.1 min, 95:5 V/V at 15.0 min; flow rate, 0.40 ml/min; temperature, 40 °C; injection volume: two μl. LIT and triple quadrupole (QQQ) scans were acquired on a triple quadrupole-linear ion trap mass spectrometer (Q TRAP), API 6500 Q TRAP LC/MS/MS System, supplied with an ESI Turbo Ion-Spray interface, running in a positive ion mode and controlled by Analyst 1.6.3 software (AB Sciex).

The ESI source operation conditions were set as: ion source, turbo spray; source temperature 500 °C; ion spray voltage (IS) 5,500 V; ion source gas I (GSI), gas II(GSII) and curtain gas were set at 65, 60 and 25.0 psi, respectively; the collision gas was high. In QQQ and LIT modes, 10 μmol/L and 100 μmol/L polypropylene glycol solutions were used to perform the tuning of the instrument and mass calibration, respectively. QQQ scans were acquired as MRM experiments with collision gas (nitrogen) set to 5 psi. DP and CE for individual MRM transitions were done with further DP and CE optimization. During the elution of metabolites in the specific period, MRM transitions corresponding to each metabolite were observed within that period (Li et al., 2018).

### Statistical analyses

For normalization, metabolite data was first Log_2_ transformed and then principal component analysis was computed using free online tool MetaboanalystR 2.0 (Chong, Yamamoto & Xia, 2019). The relative content of sugars, amino acids and organic acids were presented as Log_2_ fold change (grafted/ungrafted) whereas FPKM values of genes expression were presented as Log_2_. To visualize metabolite data and expression of the genes, the MultiExperiment Viewer software version 4.8 (http://www.tm4.org/) was used to create a heat map. Analysis of variance was performed and tested for statistical significance using Statistics 8.1. Least Significant Difference (LSD) test was used for the comparison among treatment’s means *P* < 0.05.

### RNA extraction and quality assessment

Total RNA was extracted from the flesh samples by using a Plant Total RNA Purification Kit (Gene Mark, Beijing, China) according to the product manual instructions. To evaluate the integrity, concentration and quality of RNA, an Agilent 2100 Bio analyzer (Agilent Technologies, Santa Clara, CA, USA) and a Nanodrop Nano Photometer (Implen GmbH, Germany) were used respectively.

### cDNA library preparation and sequencing

Construction of the cDNA library and sequencing were carried out at Metware (Wuhan, China). Enrichment of the mRNA with polyA tail was performed according to the method of (Godt & Roitsch, 1997) and then purified. By using N6 random primer, reverse transcription of cleaved RNA fragments to double-strand cDNA was performed. The cDNA fragments were purified, blunted with phosphate at the 5′ end and stickiness “A” at 3′ end, and adaptor-ligated. Amplification of ligated sample was completed with a pair of specific primers followed by denaturation with heat. The single-strand DNA was cyclized by splint oligo and DNA ligase. Lastly, all the cDNA libraries were subjected to Illumina HiSeq platform 4000 for sequencing (6G, 150 bp paired-end reads).

### Quality control for raw sequencing and mapping of the reads to the reference genome

The raw reads were cleaned by deleting the low-quality reads, the adaptor sequence and the unknown bases by using fastp tool (Chen et al., 2018). The Q20 and GC content for clean reads were also calculated to check the quality. After filtering, the remaining reads “the clean reads” were stored in FASTQ format (Cock et al., 2009). For the subsequent analysis, only clean reads were used. Further, clean reads were mapped to watermelon reference genome “*C. lanatus* subsp. *Vulgaris* cv. 97103 version V1” by using HISAT2 (Hierarchical Indexing for Spliced Alignment of Transcripts) software with default parameters (Kim, Langmead & Salzberg, 2015).

### Quantification of gene expression levels and screening of differentially expressed genes

A software package, “String Tie” was used to determine the expressions of genes (Pertea et al., 2015). The formula used to calculate the expression level was: FPKM = (*C*/*NL*) where *C* is the mapped fragments of transcripts, *N* represents the total counts of mapped fragments, and *L* refers to the length of the transcripts. On the basis of the raw read counts, DEseq2 R software package (http://www.bioconductor.org/packages/release/bioc/html/DESeq2.html) was adopted to identify DEGs using the following default criteria: log_2_ (fold change) > 1, and FDR < 0.05 (Love, Huber & Anders, 2014; Varet et al., 2016).

### GO term and KEGG pathway enrichment

DEGs were annotated into three core categories of GO, cellular components, biological processes, and molecular functions, using the http://www.geneontology.org/ database and “Gene Ontology” tool (Ashburner et al., 2000); The Gene Ontology Consortium (2017).

After acquiring the GO annotations for the DEGs, “Cluster Profile” an R software package was used to execute the functional enrichment analysis (Yu et al., 2012). Coordination among the genes is required to perform the particular biological event in an organism. All DEGs were categorized into the biological pathways using the Kyoto Encyclopedia of Genes and Genomes (KEGG) enrichment analysis (Kanehisa et al., 2007). Based on false discovery rates (FDR) (Benjamini & Hochberg, 1995), and using *P* < 0.05 as a threshold, significantly enriched pathways were selected.

### Validation of DEG expression by quantitative real-time polymerase chain reaction

To assess the accuracy of transcriptome data, expression analysis of nine selected DEGs was measured using quantitative real-time polymerase chain reaction (qRT-PCR) and correlation was computed with RNA-Seq data. Gene sequences were redeemed from the cucurbits genomic database (cucurbitgenomics.org).

Primer pairs were designed using the database at https://www.ncbi.nlm.nih.gov/tools/primer-blast/.

In this experiment “*Clathrin Adaptor Complex Subunit*” (*CLCAC*) was used as an internal control gene (Kong et al., 2015) (Table S1). The first-strand cDNA was constructed from one μg of total RNA, using a PrimeScript™ RT reagent kit with gDNA Eraser (Perfect Real Time) (TaKaRa, Kusatsu, Shiga, Japan) following the manufacturer’s procedure. Diluted cDNA (10 fold) was used to perform qRT-PCR analysis on the LightCycler480 RT-PCR system (Roche Diagnostics International AG, Rotkreuz, Switzerland) using LightCycler 480 SYBR Green I Master (Roche Diagnostics International AG, Rotkreuz, Switzerland) according to the manufacturer’s protocol. To carry out qRT-PCR analysis, the following conditions were followed: initial preheat at 95 °C for 5 min followed by 40 cycles of 30 s at 95 °C, 60 °C, and 72 °C. The specificity of qRT-PCR was examined using melting curve analysis at the final step of each run. Three replications were used for each experiment. Analysis of qRT-PCR raw data was performed with LCS480 v. 1.5.0.39 (Roche Diagnostics International AG, Rotkreuz, Switzerland) and the 2^−ΔΔCt^ method was used to determine the expression levels of genes (Livak & Schmittgen, 2001).

## Results

### Dynamics of SSC, pH, total sugars, total amino acids and total acid content between grafted and ungrafted watermelon

**Figure 1:**
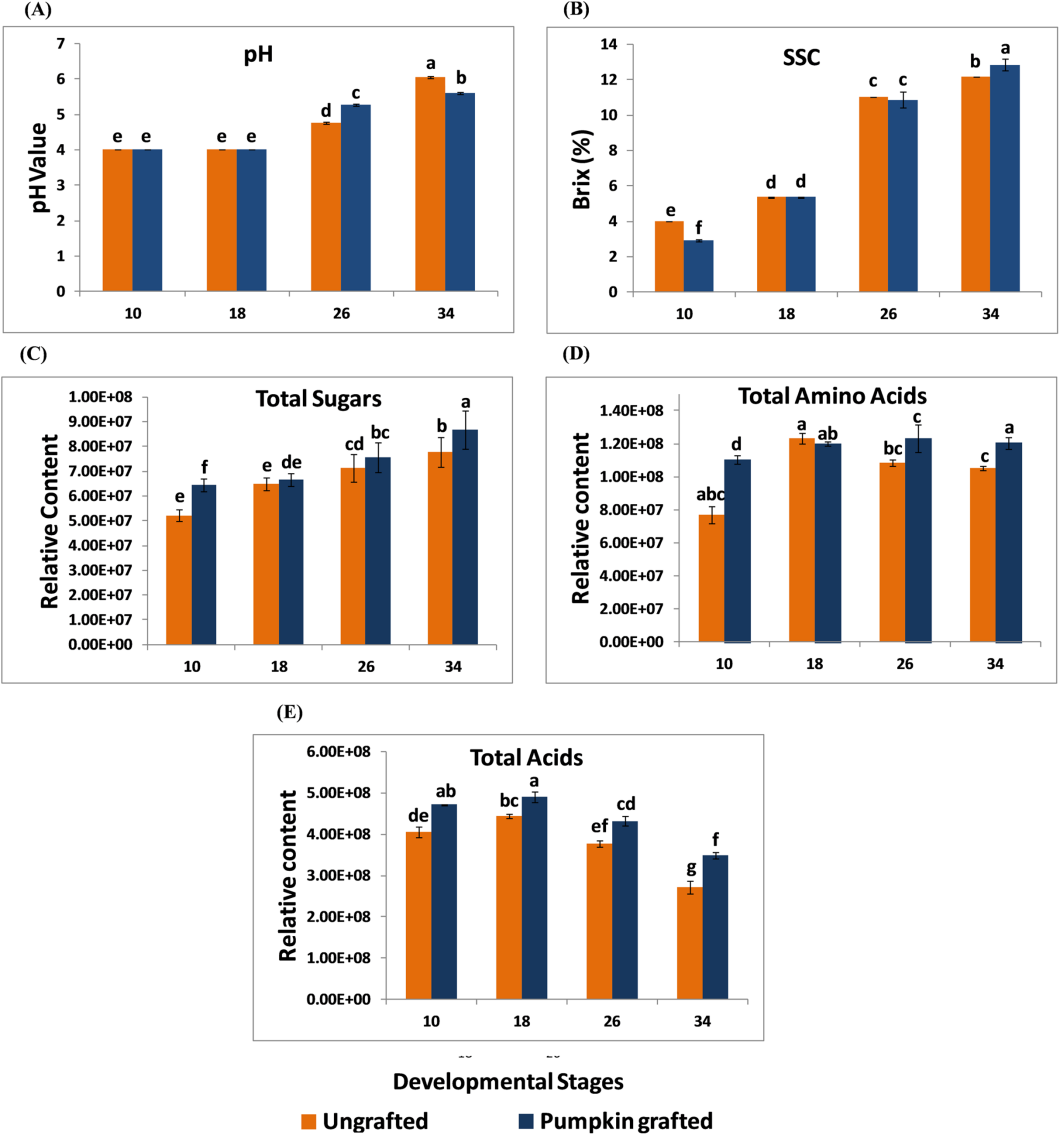
Changes in the pH (A), soluble solid contents (SSC) (B), total sugars (C), total amino acids (D) and total acids (E) in ungrafted and pumpkin-grafted watermelon during fruit development. Vertical bars represent standard error among three independent replicates. Data are the mean ± SE of three replicates. Different lowercase letters indicate significant differences at *P* < 0.05.

In grafted and ungrafted watermelon, acidity declined steadily as the fruit matured. pH was lowest at the early stages, and the highest pH was observed at later developmental stages. In addition, grafting did not influence the pH during early developmental stages while it reduced the pH at later developmental stages. Fruits from grafted watermelon were more acidic at 26 DAP as compared to ungrafted watermelon. Interestingly, acidity declined in mature fruit of 34 DAP in grafted watermelon (Fig. 1A; Table S2). SSC was significantly lower at 10 DAP in grafted watermelon fruit. From 18 to 26 DAP, SSC was relatively similar in grafted and ungrafted watermelon. Regardless of grafting, SSC progressively increases during the developmental stages, reaching the highest level at 34 DAP in grafted watermelon (Fig. 1B; Table S3). Total sugars steadily increased as the fruit matured, and were consistently higher at each developmental stage in grafted watermelon, consistent with higher SSC of mature fruit from grafted watermelon (Fig. 1C; Table S4). Total amino acids contents were found to be higher at all stages of grafted watermelon except for 18 DAP, although there was no significant difference at 18 and 26 DAP. Amino acids showed little variation among different developmental stages in both treatments (Fig. 1D; Table S5). Similar to total sugars fruits from grafted watermelon accumulated a significantly higher level of total acids at each time point. However, total acid content declined with fruit maturity in both treatments, which is consistent with the lower acidity of mature fruit (Fig. 1E; Table S6).

### Changes in sugar, amino acid and organic acid profiles of grafted watermelon

**Figure 2:**
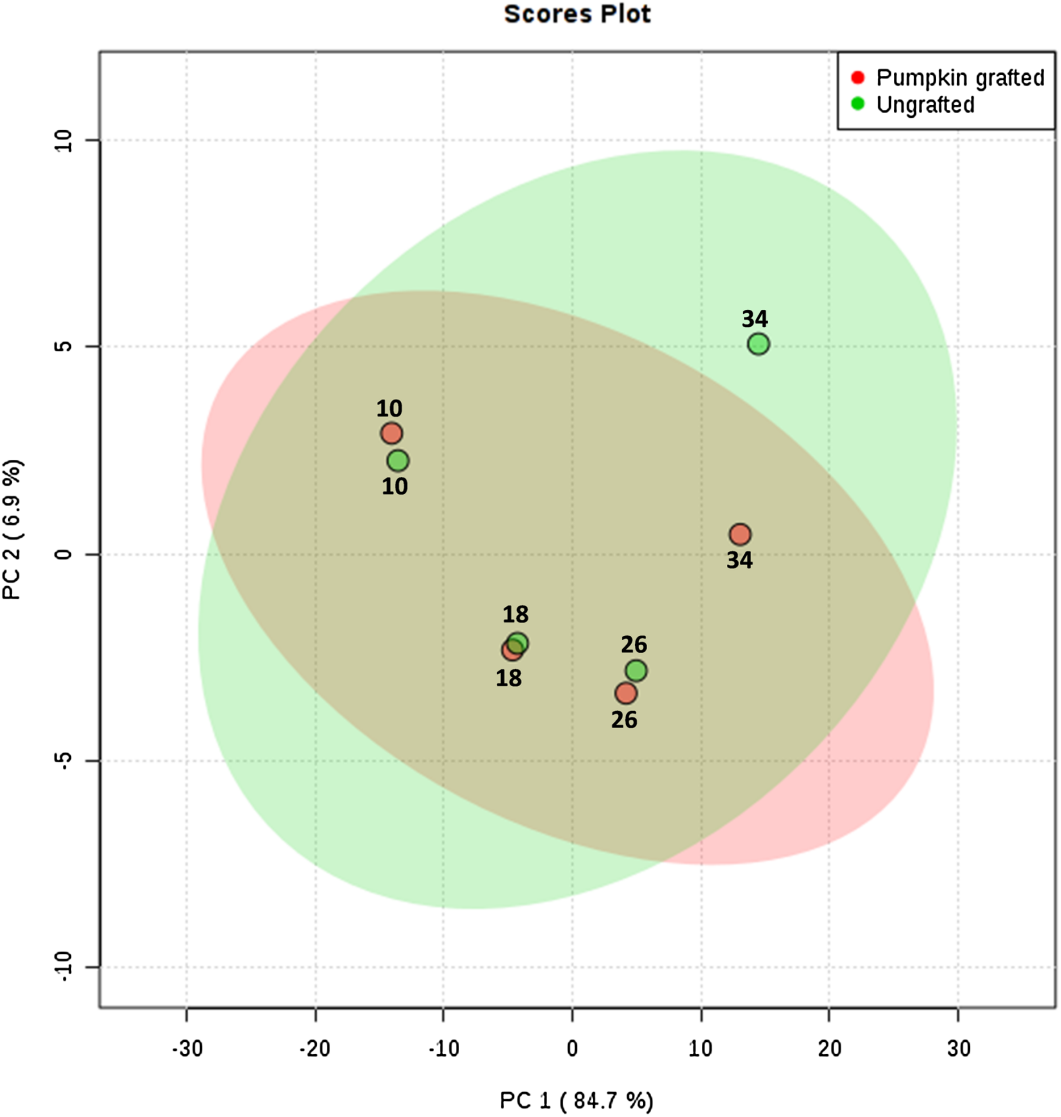
Principle component analysis: score plots for the abundance of sugars, amino acids and organic acids in ungrafted and pumpkin-grafted watermelon during fruit development. Green color represents “ungrafted,” red color represents “pumpkin-grafted.”

To interpret the changes in sugar, amino acid and organic acid profile, we performed principal component analysis. PC1 contributed to 84.7% of total variations while PC2 and PC3 exhibited 6.9% and 3.7% of the total variation, respectively. PCA analysis clearly shows the separation of metabolites at each developmental stage between grafted and ungrafted watermelon (Fig. 2; Table S7). Despite grafting, ornithine, arginine, and lysine shared the major portion of amino acids. Most of the amino acids (including L-proline, L-leucine, L-valine, L-tyrosine, L-methionine, L-asparagines, L-lysine, L-tryptophan, L-phenylalanine, L-cystein) were higher at the initial developmental stage of 10 DAP in grafted watermelon, followed by a decline at 18 DAP and then showed a peak from 26 DAP to 34 DAP. However, few amino acids showed no change or slightly higher content at immature stages (10 and 18 DAP), and then declined at later developmental stages (26 DAP and 34 DAP). Results presented herein indicate that these metabolites are not only developmentally regulated, but that rootstock has a strong impact on them (Table 1; Table S5).

**Table 1:** Represents the log2Fc (grafted/ungrafted) of the key sugars, amino acids and organic acids at 10, 18, 26 and 34 days after pollination (DAP).

Metabolites	Log_2_ of fold change (pumpkin grafted/ungrafted)			
	10	18	26	34
**Sugars**				
Sucrose	−0.82	−0.02	0.17	−0.17
D-Glucose	−0.44	0.05	−0.25	0.01
D-Trehalose	−0.93	0.19	−0.29	0.87
D-Threose	0.74	0.02	0.81	1.39
Glucosamine	1.21	−0.07	−0.21	0.27
D-Glucose 6-phosphate	0.91	0.34	0.39	0.81
d-Aminolevulinic acid hydrochloride	0.29	0.06	0.13	0.28
D-Sorbitol	−0.12	−0.04	−0.26	−0.07
Dulcitol	0.56	0.55	0.28	−1.12
**Amino acids**				
L-Arginine	0.20	0.01	0.06	−0.09
L-Alanine	0.40	0.45	0.06	−0.65
L-Valine	0.68	−0.07	0.31	1.03
L-Tyrosine	0.77	0.01	0.86	1.43
L-Asparagine	0.30	−0.06	0.01	0.04
Ornithine	0.24	0.09	0.10	0.12
L-Tryptophan	1.21	−0.81	0.33	0.74
L-Phenylalanine	0.75	−0.21	0.40	0.60
**Organic acids**				
Phosphoric acid	1.19	0.52	−0.06	0.53
2-Isopropylmalate	1.21	0.83	0.82	1.20
Succinic acid	0.05	0.27	−0.23	−0.19
Diethyl phosphate	0.30	0.29	0.60	0.12
4-Hydroxybenzoic acid	0.19	0.23	0.79	−0.03
Kynurenic acid	0.29	0.50	0.47	0.43
Azelaic acid	0.04	0.30	0.33	0.49
4-H-3-methoxymandelate	0.10	0.92	2.30	1.86
Citric acid	−0.99	−0.52	−0.23	−0.42
Citramalate	−0.09	−0.18	0.52	0.27
Terephthalic acid	0.27	0.19	0.43	0.27
A-Ketoglutaric acid	0.23	0.41	−0.15	1.33
5-h-hexanoic acid	1.05	0.89	0.67	1.46
Phthalic acid	0.27	0.21	0.56	0.71
L(−)-Malic acid	0.19	0.09	0.18	0.48
Fumaric acid	0.23	0.12	0.24	0.41

Irrespective of grafting, metabolite profiling showed that glucose, sucrose, glucosamine and D-mannose-6-phosphate were the major sugars found in watermelon. Among the sugars, the contents of dulcitol and melezitose were higher up to 26 DAP, and then showed a sudden decline at 34 DAP in grafted watermelon fruit. However, higher contents of galacturonic acid, glucopyranuronate, glucoronic acid and gala were observed only at an immature stage of 10 DAP, followed by a decline from 18 DAP to 34 DAP in grafted watermelon fruit. In addition, fruits from grafted plants exhibited a higher level of D-mannose-6-phosphate, D-glucose 6 phosphate and D-threose and delta aminolevulinic acid throughout the developmental period. Sugars including trehalose, gluconic acid, D-cellotriose, sorbitol, glucose, sucrose displayed either no change or slightly lower content at a certain time points in grafted watermelon. The levels of most of the metabolites from the glycolysis pathway were higher at mature fruit stage, which was consistent with higher SSC of grafted watermelon at mature fruit stage (Table 1; Table S4).

Despite of grafting, malic acid, fumaric acid and succinic acid were among the dominant acids. Fruits from grafted watermelon showed relatively higher contents for most of the organic acids throughout the developmental period, namely kynurenic acid, phosphoric acid, 2-isopropyl malate, diethyl phosphate, 4-hydroxy benzoic acid, 4-h-3-methoxymandelate, terephthalic acid, 5-hydroxyhexanoic acid, phthalic acid, azelaic acid, malic acid and fumaric acid. Additionally, few organic acids like (S)-2-hydroxyisocaproic acids, methyl glutaric acid, 4-guanidinobutyric acid were higher in grafted watermelon from 10 DAP to 26 DAP, and then declined at the maturity stage. The level of 2-methyl succinic acid and acetamidobutyric acid level increased, decreased and then increased again during the fruit development stages of grafted watermelon. Citramalate and pantothenic acids were slightly lower during initial developmental stages, then increased in subsequent stages of fruit development in grafted watermelon. A lower content of taurocholic acid was observed at 18 DAP, but remained stable during watermelon development, whereas butanedioldiacetate content declined at 10 and 34 DAP in grafted watermelon. A-ketoglutaric showed a distinct pattern and was higher at 10, 18 and 34 DAP in grafted watermelon. Furthermore, in grafted watermelon, citric acid and 2-benzoic acid levels were consistently low along the developmental stages in comparison to ungrafted watermelon (Table 1; Table S6).

### Summary of RNA sequence data and differentially expressed genes

Twenty-four cDNA libraries were constructed, representing four key stages of fruit development. We used three replicates for each stage and treatment. Low-quality reads (reads containing an adapter, reads containing poly-N, and reads with Qphred ≤20 equivalent to reads with base call accuracy less than 99%) were eliminated to obtain the clean data (high-quality clean reads). RNA sequence data from all the samples showed that >90% reads were uniquely mapped to the reference genome, and at the Q20 level, 96% of clean data had a Phred-like quality score. High-quality RNA sequence data laid the foundation for identification of genes potentially involved in the metabolism of sugars, organic acids and amino acids. A summary of RNA sequencing data is presented in Table 2. All the raw data of RNA-Seq has been submitted to NCBI Sequence Read Archive having Accession Number “PRJNA543725.”

**Table 2:** Summarized results of RNA-seq data. *N* denotes ungrafted watermelon. *P* denotes grafted watermelon. 10, 18, 26, 34 represents days after pollination (DAP). I, 2, 3 indicate replications. Total Mapped Reads (%) = Unique Match (%) + Multi-position Match (%), are the percentages of clean reads align to reference genome. Q20 (%) are the percentages of reads with Phred qualities scores over 20.

Sample	Raw reads number	Clean reads number	Total mapped reads (%)	Uniquely mapped reads (%)	Multiple mapped reads (%)	Q20 (%)	GC content
N10-1	25574730	50758156	96.85	94.49	2.36	97.98	43.895
N10-2	26610172	43942374	96.51	94.10	2.41	96.775	44.485
N10-3	28050313	42829148	96.52	93.99	2.53	97.48	44.285
N18-1	25514128	52072666	96.79	94.04	2.75	97.78	44.295
N18-2	27252032	51176968	96.51	93.83	2.68	97.78	44.42
N18-3	28486773	69912288	96.68	93.92	2.76	97.51	44.205
N26-1	28384626	47641598	96.61	93.98	2.63	97.98	43.97
N26-2	26378372	58006502	96.66	93.61	3.05	97.985	44.075
N26-3	25397575	54678486	96.87	93.99	2.88	97.84	44.215
N34-1	31255473	53671042	96.88	94.21	2.67	97.94	43.95
N34-2	25384004	55309080	96.65	94.10	2.55	97.755	43.82
N34-3	24800859	52739582	97.11	94.45	2.66	98.02	43.97
P10-1	25943842	49887194	96.38	93.90	2.48	97.515	44.335
P10-2	22461775	52017420	96.64	94.07	2.57	97.47	44.325
P10-3	22031430	54781854	96.50	94.16	2.34	97.545	43.735
P18-1	26741188	49788246	96.94	94.13	2.81	98.085	44.37
P18-2	26571966	52685694	96.50	93.92	2.58	97.55	44.18
P18-3	36355949	54885196	96.73	94.11	2.62	97.86	44.225
P26-1	24566638	55331688	97.08	94.30	2.78	97.865	44.16
P26-2	29870856	51366352	96.95	94.16	2.79	97.855	44.115
P26-3	28425395	49070004	96.59	93.71	2.88	97.99	44.1
P34-1	27666791	61328702	96.91	94.02	2.89	97.97	44.075
P34-2	28438527	49831596	96.66	93.86	2.80	97.56	44.01
P34-3	27014139	48431750	96.51	93.85	2.66	97.6	43.87

**Figure 3:**
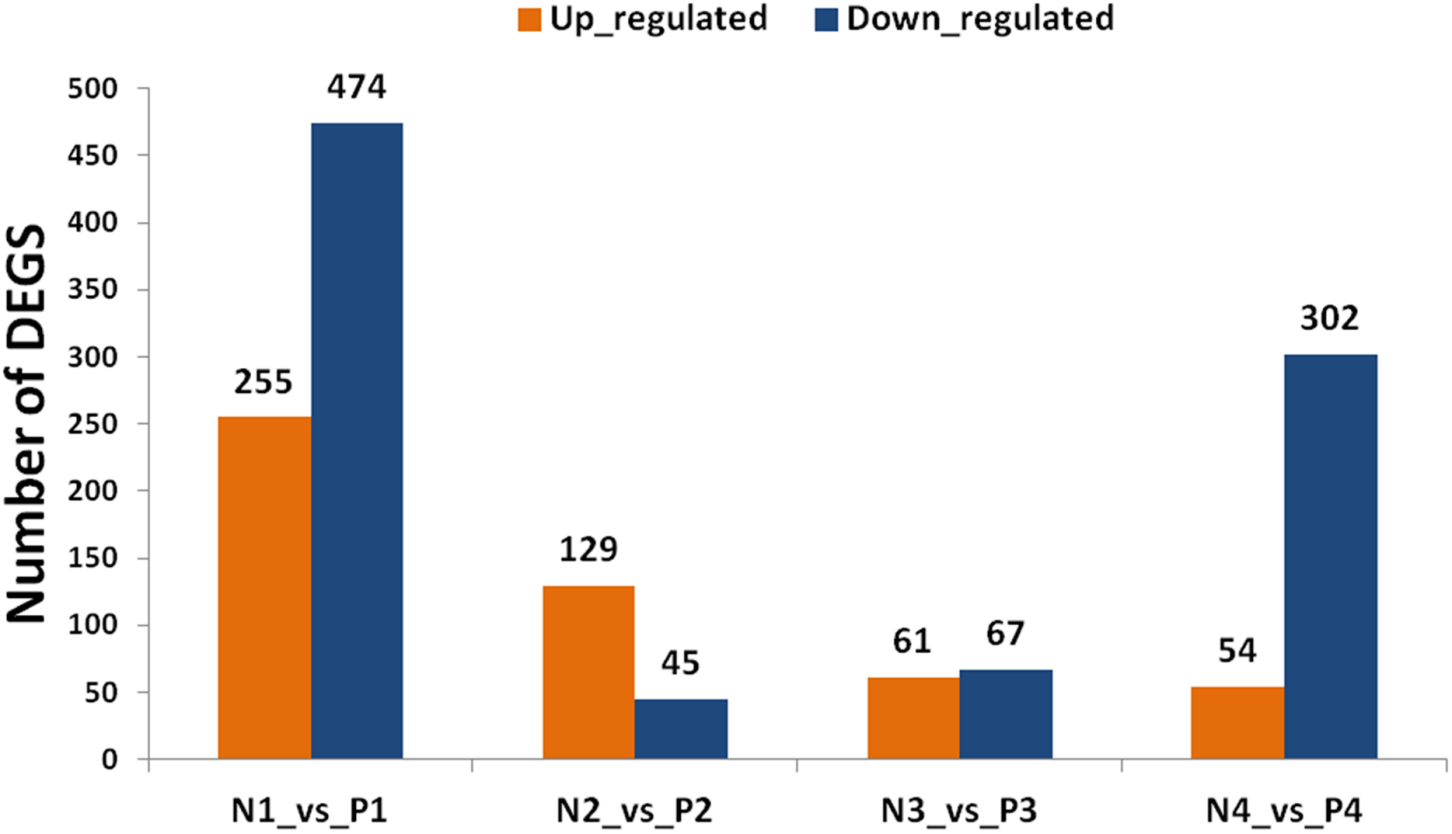
Classification of differentially expressed genes (DEGs). *X* axis shows number of DEGs and *Y* axis represents number of DEGs in pair wise comparison. N1_vs._P1, N2_vs._P2, N3_vs._P3 and N4_vs._P4 indicate comparison between ungrafted and pumpkin-grafted watermelon fruit at 10, 18, 26 and 34 days after pollination (DAP) respectively. Blue bar denotes down-regulated genes and orange bar for the up-regulated genes.

By using the criterion of FDR < 0.05 and log_2_ fold >1 as a threshold, DEGs were screened out between the two samples for further functional analysis (Table S8). Comparisons of “ungrafted” and “grafted” watermelon at the same developmental stages indicated there were 729 (P1 vs. N1) and 356 (P4 vs. N4) DEGs. In contrast, few DEGs were detected at P2 vs. N2 and P3 vs. N3, accounting for 174 and 128 DEGs, respectively (Fig. 3). Furthermore, during all developmental stages, the number of up-regulated DEGs in ungrafted vs. grafted samples were lower as compared to down-regulated DEGs.

Genes sharing common functions often expressed at the same level. For that reason, genes were clustered hierarchically. Comparison of developmental stages within the same material, such as pumpkin-grafted watermelon where P1 was the control, and subsequent stages discloses that the number of DEGs were higher in P1 vs. P4 in comparison to P1 vs. P3 or P1 vs. P2. Similar results were observed in ungrafted watermelon.

The results showed that the number of up and down-regulated genes were higher in P1 vs. P4 and N1 vs. N4 as compared to other groups. Number of DEGs were low at 10 DAP, and their number began to increase as the fruit continues to develop in order to participate in complex regulatory mechanisms (Fig. S2).

Comparison of two materials at the same developmental stages yielded a higher number of DEGs, and even far greater number of DEGs were found among the different developmental stages within the same material indicating complex regulatory mechanisms could be involved in fruit quality.

### Significantly altered gene ontology terms and chemical pathways in grafted watermelon

**Figure 4:**
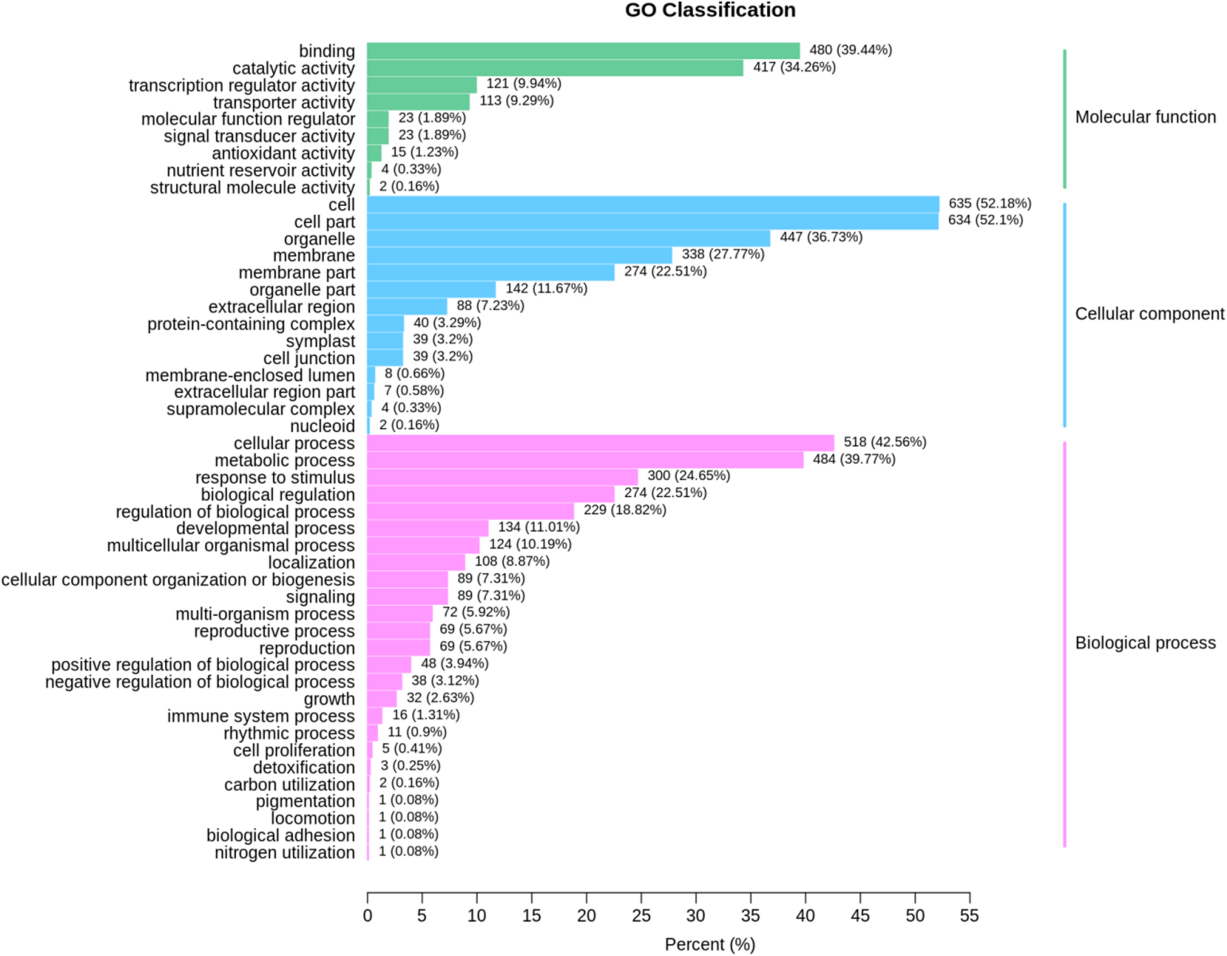
GO classification for the differentially expressed genes (DEGs) in all sample comparison. *X* axis shows percentage of DEGs. *Y* axis indicates GO terms. All GO terms are classified into three categories: Blue, green and pink colors represent cellular component, molecular function and biological process, respectively.

Based on GO term analysis, three functional categories namely biological process, cellular component, and molecular function were assigned to DEGs that were identified at the same developmental stage between ungrafted and grafted watermelon. GO enrichment results are presented in Fig. 4; Table S9. In the GO category of “biological process,” highly represented GO terms were “metabolic process” and “cellular process” whereas “cell,” “cell part” and “organelle” were frequent in the “cellular component” category. In the GO category of “molecular function,” “catalytic activity,” “binding” and “transporter activity” were also frequent.

Many of these genes were involved in primary processes associated with fruit quality, such as amino acids and sugars, suggesting the high impact of rootstock on the metabolic processes related to fruit quality.

**Figure 5:**
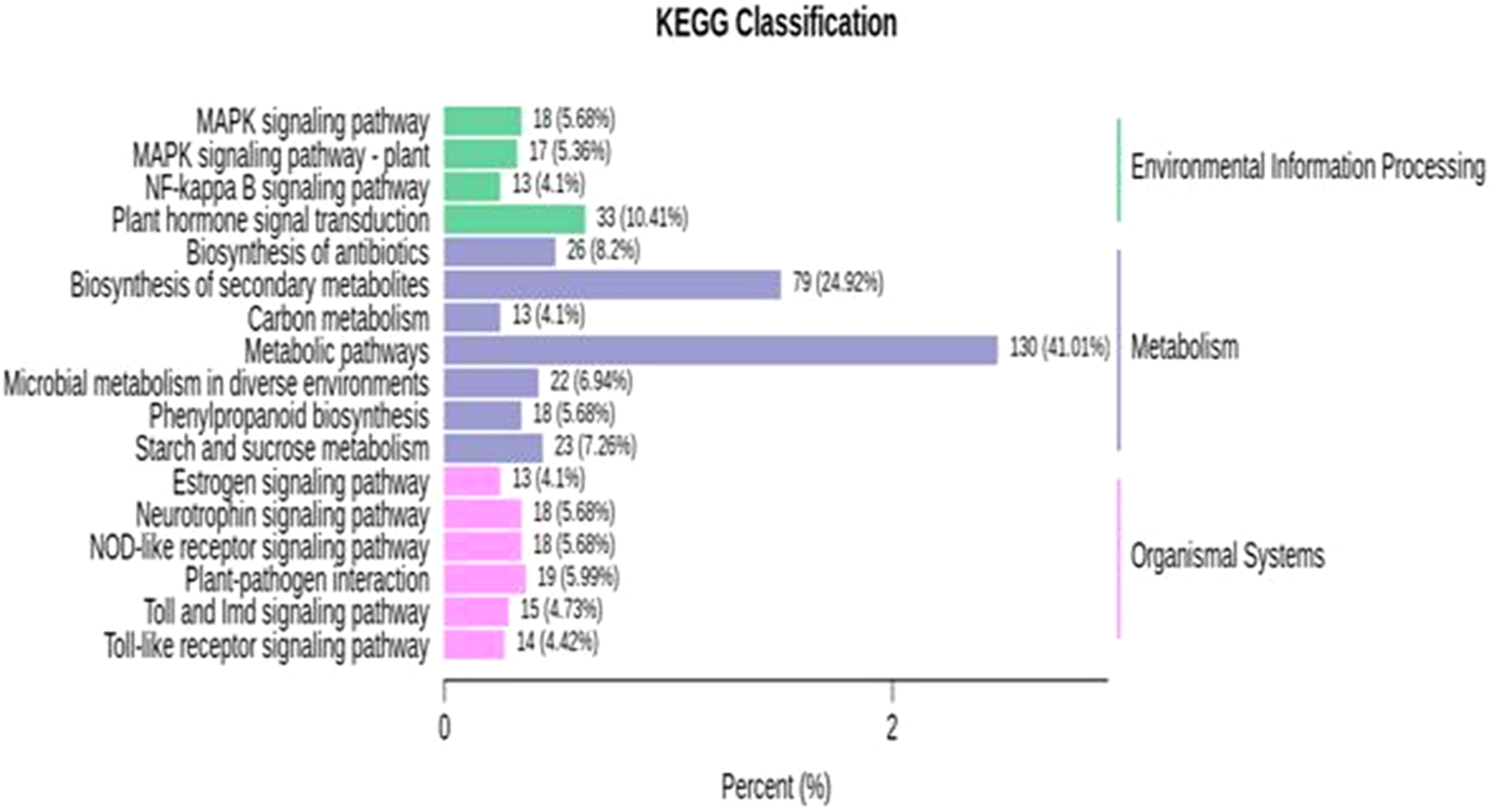
KEGG enrichment analysis for differentially expressed genes (DEGs) in all sample comparison. *X* axis means number of DEGs. *Y* axis represents KEGG pathway terms. All pathway terms are grouped in top pathway terms indicated in different color.

KEGG enrichment analysis was performed for identified DEGs. For the further identification of biological pathways, DEGs were mapped to the reference pathways using the KEGG database and were compared to the whole transcriptome background. From the transcriptome analysis, we identified 138 (531 DEGs), 89 (136), 97 (130), 139 (427) pathways at 10, 18, 26 and 34 DAP, respectively. Based on enrichment level and the number of annotated DEGs, significantly enriched pathways were selected and are presented in Fig. 5; Table S10. The over-represented pathway includes plant hormone signal transduction, signaling pathway, plant-pathogen interaction, metabolic, starch and sucrose metabolism, carbon metabolism and phenylpropanoid pathway. The results demonstrate that grafting plays a crucial role in modulating the expression of genes related to fruit quality during the development of fruit. The transcriptome analysis provides a great opportunity for understanding the metabolic process largely driven by grafting during the development of watermelon.

### Graft responsive DEGs involved in sugar metabolism

The accumulation of metabolites demonstrates changes in fruit quality largely driven by grafting. To gain deeper insight into the molecular mechanism involved in the metabolism of main sugars, major organic acids, and amino acids, we performed pairwise comparison between ungrafted and grafted watermelon at each developmental stage.

**Figure 6:**
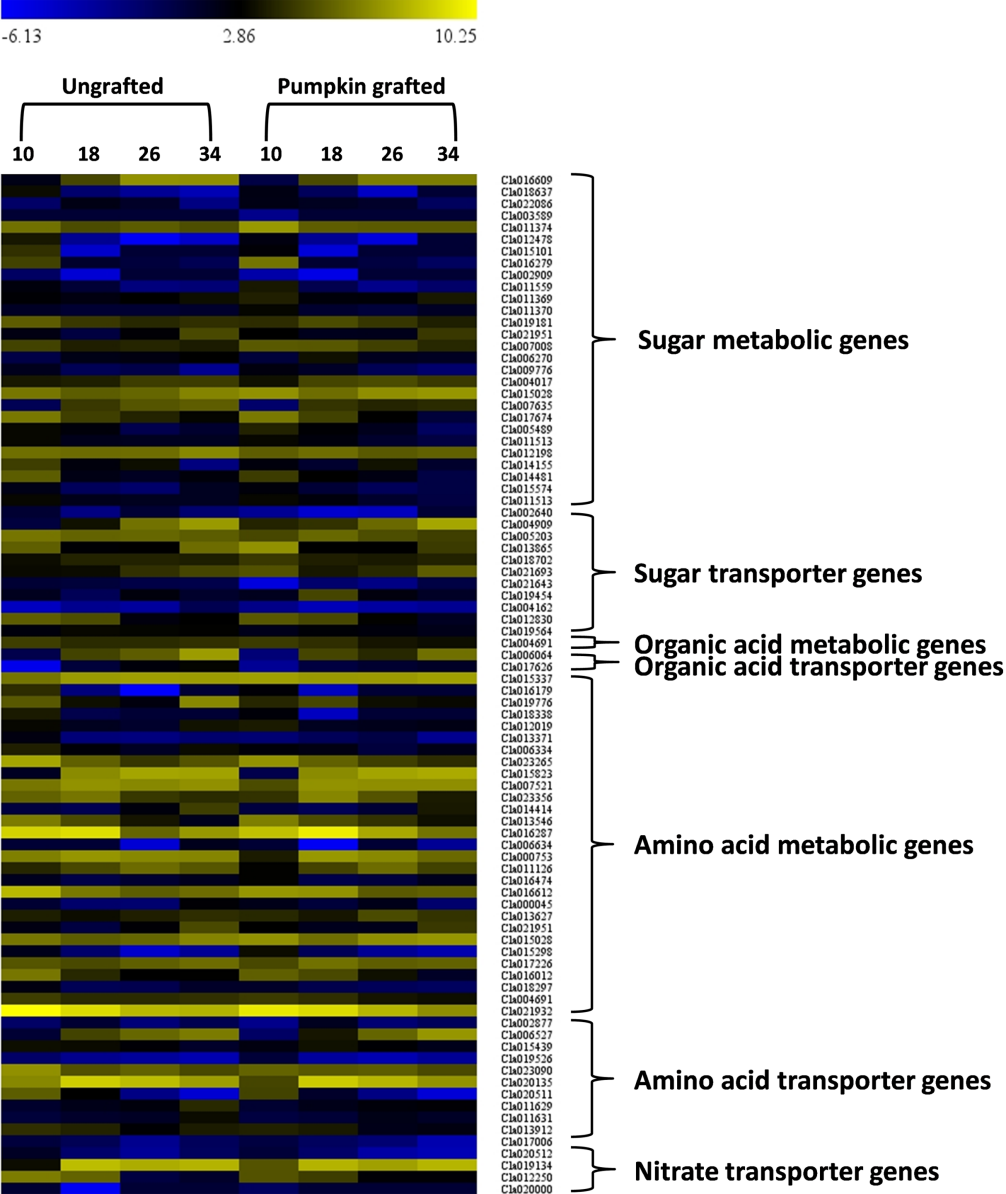
Heat map represents expression values (log2 FPKM) of differentially expressed genes in at least one of the developmental stage comparisons of ungrafted vs. pumpkin-grafted watermelon. Ten, 18, 26 and 34 indicate days after pollination (DAP). FPKM value plotted in each cell is the average of three replicates. Red color shows high expression level, black color shows middle value and green color shows low expression value.

In the sugar metabolic pathways (glycolysis, starch and sucrose, fructose and mannose), 28 genes showed differential gene expression at different development stages (Fig. 6; Table S11). Among the DEGs, six genes encoding *fructose-bisphosphate aldolase 2 (FBA2)(Cla016609), fruckto kinase (Cla007008), sucrose synthase (SuSy) (Cla018637), sucrose-phosphate synthase (SPS) (Cla012198), insoluble acid invertase (IAI) (Cla017674) and invertase (Cla011559*) showed distinct patterns in grafted watermelon fruit, consistent with sugar phenotype. Expression of *Cla016609 enocidng FBA2* progressively increased along the developmental stages, but expression was lower in grafted watermelon fruit. *Cla007008* encoding *fructokinase* (*FK)* were down-regulated during development, and expression was higher in grafted watermelon fruit. Consistent downregulation of genes encoding *SuSy (Cla018637)* was noticed during fruit development in grafted and ungrafted plant, but grafted plants tended to have lower transcript abundance of *SuSy*. *Cla012198* encoding *SPS* was highly down-regulated at the mature stage in grafted watermelon. Two genes annotated as *IAI (Cla017674)*, and acid invertase *(Cla011559)* were also down-regulated along developmental stages in grafted watermelon. Although the expression of *IAI* was relatively similar during earlier fruit development, it was reduced in subsequent developmental stages of grafted watermelon fruit. However, acid invertase (*AI*) tended to have lower expression during the middle of fruit development while its expression was higher at 10 DAP and 34 DAP.

### Graft responsive DEGs involved in organic acid metabolism

In the citrate cycle, we detected one differentially expressed gene, *Cla004691*, that encodes *2-oxoglutarate dehydrogenase* (*OGDH*) and exhibits reduced expression in later stages of grafted watermelon fruit that positively correlated with lower citrate and succinic acid content (Fig. 6; Table S11).

### Graft responsive DEGs involved in amino acid metabolism

We further assessed the amino acid metabolic pathways and identified 29 DEGs associated with amino acid metabolism. Among DEGs, 10 genes were highly correlated with various amino acid contents (Fig. 6; Table S11). For example, in glutamine, ornithine, arginine and citrulline metabolic pathway, *acetylornithine deacetylase (NAOD, Cla016179)* differentially expressed during development and showed consistently lower expression in grafted watermelon, which negatively correlated with ornithine content. However, it showed a positive correlation with citrulline content, and significant down-regulation of *glutamine synthetase (GS, Cla188338)* at earlier fruit developmental stages coincides with higher glutamine content.

In the metabolic pathway of alanine, aspartate and glutamate, *Cla006334* encoding *alanine-glyoxylate aminotransferase* (*AGT*) was down-regulated at 34 DAP. This correlates with lower alanine content at this time point, and from 10 to 26 its expression was interestingly higher in grafted watermelon, which was consistent with higher alanine content. In the biosynthetic pathway of tyrosine, one DEG annotated as *tyrosine aminotransferase (TaT, Cla013546)*. It was down-regulated with development and showed a similar expression pattern in grafted and ungrafted watermelon, but its expression was consistently higher in grafted watermelon, consistent with higher tyrosine content. Another gene, *Cla015028* encoding *alcohol dehydrogenase* (*aDH1*), mapped to the tyrosine pathway and was found to be up-regulated during the development of grafted watermelon.

*Cla004691* encoding *2-oxoglutarate dehydrogenase* involved in the degradation of lysine only showed slightly higher expression at 18 DAP, leading to lower lysine content at this time point in grafted watermelon. In the metabolic pathway of arginine and proline, *Cla016612* was annotated as *arginine decarboxylase* (*aDC*) and was differentially expressed during fruit ripening. Expression was only higher at 18 DAP, which coincides with lower arginine content. In the pathway leading to biosynthesis of phenylalanine, lower phenylalanine content was associated with higher expression of *Cla017226* and *Cla018297* encoding *4-coumarate--CoA ligase 1* (*4CL 1*) and *Phenylalanine ammonia-lyase* (*PaL*), respectively, at 18 DAP. Only one differentially expressed gene annotated as *catalase isozyme 3 (CaT, Cla02193)* in the tryptophan metabolic pathway was significantly downregulated with fruit development and showed a similar expression trend in grafted and ungrafted watermelon. However, expression was lower in grafted watermelon (Fig. 6; Table S11).

### Graft responsive transporter genes encoded by DEGs

Signaling and transport across the graft union is a key event controlling scion growth and fruit quality (Notaguchi, Higashiyama & Suzuki, 2014). In this study, we screened out DEGs coding for transporter proteins related to sugars, amino acids and organic acids. A total of 63 transporter genes differentially expressed at least one of the developmental stages. Among them, two were classified as aluminum-activated malate transporters (*ALMT13* and *ALMT8*), 13 belonged to amino acids and 11 were related to sugar transport (Fig. 6; Table S11). Interestingly, two organic acid transporters were differentially expressed at 26 and 34 DAP and were highly down-regulated. Generally, most of the amino acid and sugar transporters were down-regulated in grafted watermelon during fruit developmental stages. However, we also detected transporter genes related to copper nitrate, phosphate, sulfate, zinc, nitrite, folate, oligopeptide, ascorbate, metal-nicotianamine, molybdate, potassium, vacuolar iron transporter, urea, xylose, boron and ABC, which may play regulatory role in modulating fruit quality (Table S12).

### Validation of differential gene expression data by qRT-PCR

**Figure 7:**
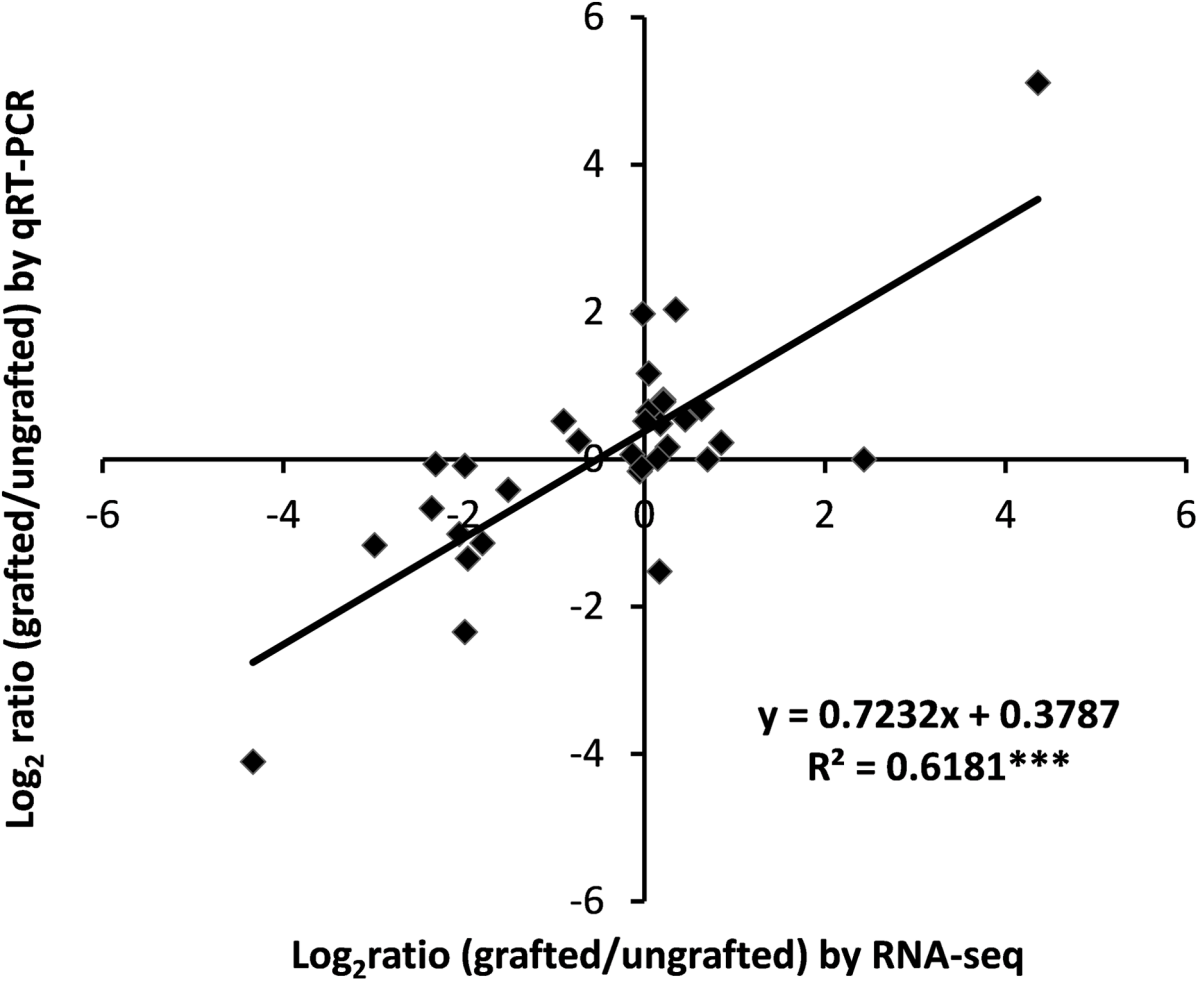
Correlation analysis of qRT-PCR and RNA-Seq data.

To assess the accuracy of transcriptome data, expression analysis of nine selected DEGs was measured using qRT-PCR and correlation was computed with RNA-Seq data. Results showed a highly significant correlation of qRT-PCR results with RNA-Seq output (Fig. 7; Table S13).

### Discussions

Grafting has long been practiced to combat against soil borne pathogens and diseases, and to enhance plant resistance against stresses (Miguel et al., 2004). Grafting has been shown to improve fruit quality, yield and nutrient uptake (Rouphael et al., 2010). The role of grafting in mediating the comprehensive metabolic profiling and underlying molecular mechanism has never been reported in watermelon. This study will present the differences in the primary metabolomic profile and regulatory mechanism in ungrafted and pumpkin-grafted watermelon.

### Effect of grafting on pH, total soluble solids, total sugars, total amino acid and total acid content

Grafting influences fruit quality (Bletsos & Passam, 2010). Acidity decreases as fruits develop, and at early stages, no change in pH is noticed between grafted and ungrafted watermelon. At 26 DAP, grafted plants produce more acidic fruit, but at the ripe stage, fruits become less acidic, when compared with ungrafted watermelon, which agrees with previous findings (Petropoulos et al., 2014; Proietti et al., 2008; Rouphael et al., 2010). Our results are in line with previous studies reporting that TSS tends to rise as fruit develops and grafting positively influences the TSS of mature watermelon fruit (Mohamed et al., 2014). Total sugars were consistently higher in grafted watermelon, which correlates with higher SSC. Higher total sugar contents were reported in ripe watermelon fruit grafted onto commercial bottle gourd rootstock CT/33-45 (Çandir et al., 2013). Total organic acids were consistently higher in grafted watermelon, while main organic acids gradually decline with development, which is consistent with previous findings where main organic acid content in sand pear fruits declined with fruit development (Huo et al., 2009). Total amino acid content was higher in grafted watermelon during the developmental stages except at 18 DAP. Similar results were reported in mature grapes that grafting enhanced amino acid content (Kubota, Li & Yasui, 1993).

### Effect of grafting on sugar, amino acid and organic acid metabolic profiles

Glucose and fructose were found to be dominant sugars in immature white watermelon, and their level begin to decline as fruit develops. In contrast, sucrose was lowest at the immature stage, followed by a sharp increase as the fruit develops, and was dominant in mature watermelon (Guo et al., 2011). Our results showed that glucose and sucrose were the dominant sugars, and sucrose was the predominant sugar in mature grafted watermelon. Sugars including trehalose, sucrose, glucose, cellotriose and sorbitol showed no change or little variation during the development of grafted watermelon, which is concurrent with the findings of Proietti et al. (2008).

In grafted watermelon, other sugars like D-mannose 6 phosphate, D-glucose 6 phosphate and D-threose, delta aminolevulinic increased during the fruit developmental stages. While glucoronic acid, gala, D-glucopyranuronate and gluconic acid decreased from 18 DAP to 34 DAP. Moreover, contents of melezitose and dulcitol declined only at the mature stage. Grafting influences endogenous production of secondary and primary metabolites, the positive and negative impacts of grafting on fruit quality have been reported by Alexopoulos, Kondylis & Passam (2007), Liu et al. (2016) and Tsaballa et al. (2013).

In watermelon, citric acid and malic acids are key organic acids (Gao et al., 2018). Our results suggested that malic acid, fumaric acid, succinic acid and citramalate are dominant acids produced during the citrate cycle. Differences in the results may be attributed to the use of different varieties. Most importantly earlier studies measured a limited number of acids. Most of the organic acids relatively increased, and few declined only at maturity. Citric acid and 2 benzoic acid displayed lower contents throughout the development of grafted watermelon fruit, suggesting the high impact of rootstock in modulating the acidic profile of watermelon flesh. Higher malic acid and reduction of citric acid content during developmental stages of grafted watermelon was in agreement with previous studies (Fredes et al., 2017).

Amino acids vary with crops and impart different flavors to fruit. In this study, ornithine, arginine, lysine, glutamine and tyrosine were the major amino acids similar to grapes (Kubota, Li & Yasui, 1993) and muskmelon (Liu et al., 2010). Most of the amino acids accumulated in higher amounts in grafted watermelon during fruit development except at18 DAP. Specifically, glutamic acid content markedly increased from 10 DAP to 26 DAP. Plants use glutamic acid as a nitrogen source for the biosynthesis of nitrogenous compounds. Similar results were reported by Hammond-Kosack & Jones (2000) and Zhang, Liu & Ruan (2017).

### Regulation of sugar metabolic genes in grafted watermelon fruit

Three enzyme families, namely *insoluble acid invertases* (*IAI*), *sucrose synthases* (*SuSy*), *SPSs*, have been implicated in the determination of sugars in watermelon fruit (Liu et al., 2013; Yativ, Harary & Wolf, 2010). *SuSy* is regarded as an important enzyme in sucrose metabolism, catalyzing the biosynthesis and breakdown of sucrose in plants. *SuSy (Cla018637)* was down-regulated during fruit development in “grafted” and “ungrafted” watermelon, Sucrose content showed an inverse correlation with transcript abundance of *SuSy (Cla018637)*. Our results are not in agreement with earlier studies, which reported the positive correlation of sucrose content with *SuSy* genes (Liu et al., 2013; Zhu et al., 2017). The disparity in the reported result might be due to the differential role of different *SuSy* genes involved in sucrose metabolism and accumulation, and processes are reversible (Gao et al., 2018). *SPSs* catalyze sucrose synthesis and showed a positive correlation with sucrose level in melon (Hubbard, Huber & Pharr, 1989), tomato (Dali, Michaud & Yelle, 1992) and watermelon (Zhu et al., 2017). In our study, *SPSs* (*Cla012198*) showed high down-regulation at the mature fruit stage of grafted watermelon, consistent with lower sucrose content. The process where gene expression is not reflected in phenotype change suggests that a posttranslational modification may be involved in controlling *SPS* enzyme activity (Zhao et al., 2018).

Translocation of sucrose to the fruit peel and the unloading to phloem were attributed to *insoluble acid invertase* activity in plants like tomato (Godt & Roitsch, 1997), carrot (Tang, Lüscher & Sturm, 1999) and watermelon (Liu et al., 2013). *Cla017674* belongs to an ortholog of *invertase* associated with sucrose content, which was constantly down-regulated in both treatments and its expression was significantly lower in grafted watermelon fruit from 26 DAP to 34 DAP, suggesting the involvement of *Cla017674* in extracellular sucrose breakdown (Gao et al., 2018). In contrast, *Cla011559* encodes *soluble invertases*, found to be up-regulated at 10 DAP and 34 DAP in grafted watermelon, which is consistent with the lower sucrose content at these time points. Previously, *low soluble invertase* activity was predicted in high sucrose-accumulating watermelon cultivars (Yativ, Harary & Wolf, 2010). Involvement of multiple genes in sucrose metabolism suggests a complex regulatory mechanism controlling sucrose accumulation (Koch, Wu & Xu, 1996).

*FBA2* plays a central role in the glycolysis pathway and is associated with glucose (Lv et al., 2017). Expression of *FBA2* was relatively lower during fruit developmental stages in grafted watermelon, which correlates with lower or stable glucose content in grafted watermelon. FK is likely to be involved in the phosphorylation, thereby influencing the glycolysis/gluconeogenesis pathway (Granot, David-Schwartz & Kelly, 2013). *Cla007008* encoding *fructose kinase* (*FK*) was shown to have higher expression in grafted watermelon at 18 DAP than at other stages. Studies have shown that citric acid might prevent the synthesis of the FK enzyme and could enhance ATP production (Papagianni, Avramidis & Filiousis, 2007). Citric acid declines as a result of grafting, which may alleviate effects on the enzyme and increase the transcript level.

A total of 11 sugar transporter genes differentially expressed and regulated during fruit development in grafted watermelon. Most of the sugar transporter genes were down-regulated in grafted watermelon during fruit development. Notably, one *SWEET like sugar* (*SWT3b) Cla004909* transporter gene was identified and highly expressed in grafted watermelon. This result indicates the roles of these transporter genes in active membrane transport of sugars influencing the sugar content of watermelon fruit (Guo et al., 2015).

### Regulation of organic acid metabolic genes in grafted watermelon fruit

Gene *Cla004691* encoding *2-oxoglutarate dehydrogenase* (*OGDH*) was mapped to the citrate pathway, which showed similar expression in both treatments at early fruit developmental stages. It was down regulated in the mature fruit of grafted watermelon and positively correlated with citrate content. Araújo et al. (2012) reported higher citrate and isocitrate content, lower fumarate and malic acid content in transgenic tomato lines deficient in the expression of *OGDH*, suggesting a different mode of activity in different crops. Notably, various studies have also reported the insignificant correlation between organic acid contents and metabolic genes (Etienne et al., 2002; Moing et al., 2000; Yao et al., 2009).

Furthermore, an *aluminum-activated malate transporter* (*ALMT*) has been predicted to be involved in vacuolar malate transport and accumulation in apple (Bai et al., 2012) and tomato (Ye et al., 2017). *Cla006064* and *Cla017626* are the orthologs of *ALMT* that were down-regulated during fruit developmental stages in grafted watermelon, correlating with malic acid accumulation. The results mentioned above suggest that these genes may play a central role in malic acid accumulation by reducing malic acid transport into the vacuole during ripening process (Gao et al., 2018).

### Regulation of amino acid metabolic genes in grafted watermelon fruit

Most of the amino acid contents increased as a consequence of grafting in watermelon during fruit development and ripening except at 18 DAP. A few amino acids declined only at maturity. Among DEGs, 10 genes were highly correlated with various amino acid contents. For example, in the glutamine, ornithine, arginine and citrulline metabolic pathways, high down-regulation of *acetylornithine deacetylase* (*Cla016179*) negatively correlated with ornithine content in grafted watermelon. In the final step of the ornithine pathway, acetylornithine deacetylase (*NAOD*) synthesized ornithine by releasing acetate. Down-regulation of *NAOD* was concurrent with reduced level of ornithine in *AtNAOD*-silenced and T-DNA insertional mutant (*AtNAOD*) plants of Arabidopsis (Molesini et al., 2015). Results suggesting that *NAOD* is of high importance in ornithine cycle and can regulate ornithine synthesis. Significant down-regulation of glutamine synthetase at earlier fruit developmental stages coincided with higher glutamine content. Inhibition of *glutamine synthetase* by phosphinothricin has been reported to suppress asparagine biosynthesis and depletion of glutamine in *Medicago truncatula* indicating its key role in regulating biosynthesis of glutamate (Seabra et al., 2012).

In the metabolic pathway of alanine, aspartate and glutamate, *alanine aminotransferase* catalyzes the reversible transamination between alanine and 2-oxoglutarate to form pyruvate and glutamate (Xu et al., 2017). *Cla006334* encoding *alanine-glyoxylate aminotransferase* was higher from 10 DAP to 26 DAP and then was highly down regulated at 34 DAP in grafted watermelon, which aligns with the changes in alanine content. A parallel increase in alanine content upon induction of the expression of *alanine aminotransferase* during flooding has previously been documented (Muench & Good, 1994). *Cla006334* might have led to decreased transamination of alanine to glutamate and pyruvate thus increasing the alanine content.

In the biosynthetic pathway of tyrosine, *TaT (Cla013546)* and *aDH1 (Cla015028)* were higher in the grafted watermelon, consistent with higher tyrosine content. *TaT* knock out mutant of microbe has been shown to be deficient in tyrosine (Umbarger, 1978). *TaT* is involved in the first step of tyrosine metabolism our results shows that *TaT* might be critical for biosynthesis of tyrosine. In the lysine degradation pathway, a lower transcript level of gene encoding *2-oxoglutarate dehydrogenase (Cla004691)* at 10, 26 and 34 DAP enhanced valine, asparagine, and phenylalanine contents at these time points. Amino acids such as valine, isoleucine, glutamate, asparagines and phenylalanine were higher in *OGDH* mutant lines than that of wild type of tomato. *2-oxoglutarate dehydrogenase* (*OGDH*) acts as a point of junction that regulates the flux from oxoglutarate to amino acid synthesis showing its connecting role in controlling the biosynthesis of oxoglutarate derived amino acids (Araújo et al., 2012).

In the metabolic pathways of arginine and proline, *Cla016612* encoding *(aDC)* showed high expression at 18 DAP in pumpkin-grafted watermelon. Polyamine biosynthesis in Arabidopsis begins with arginine, which is converted by *aDC*. Higher transcript abundance might have led to an increased break down of arginine into polyamines thus decreasing its content (Bagni & Tassoni, 2001). In the phenylalanine pathway, a lower phenylalanine content at 18 DAP was associated with a higher expression of *4CL 1 (Cla017226)* and *PaL (Cla018297)*. Similar results were reported in grafted grapes (Marè et al., 2013). Phenylalanine ammonia lyase is involved in the conversion of phenylalanine into trans-cinnamic acid (Scheible et al., 2004). Lower phenylalanine content might have resulted due to higher breakdown of phenylalanine into trans-cinnamic acid which led to reduce phenylalanine content.

Previously, multiple roles have been reported for *catalase* other than amino acids metabolism (Hu et al., 2010). In the tryptophan metabolic pathway, *catalase isozyme 3 (Cla021932)* showed reduced expression in grafted watermelon, suggesting its role in the degradation that enhances the accumulation of tryptophan. Amino acid transporters (AATs) mediate the transport of amino acids across cellular membranes in higher plants, long-distance transport, and response to biotic and abiotic stresses (Tegeder, 2012). Interestingly, all the amino acid transporter genes were down-regulated at various developmental stages suggesting that the accumulation of amino acids are governed by graft-responsive metabolic genes.

Additionally, our results were further endorsed by nitrate transporter genes which showed up-regulation at all developmental stages, except at18 DAP. Expressions of *nitrate transporter 1.10* (*NRT1, Cla019134*) and *nitrate transporter 1.4* (*NRT1, Cla012250*) were consistent with higher amino acid content. Enhanced expression of nitrate transporter has been reported in grafted tomato (Albornoz et al., 2018). The grafting-induced changes may be due to the process of grafting itself or the impact of heterografting to a pumpkin rootstock or that can also be attributed to changed soil conditions due to continuous watermelon cultivation in same field which warrants further investigation in self-grafted and in other rootstocks. It is possible that the transcriptional changes observed in our dataset are due to the transfer of some mobile substances, such as hormones or various RNA species, across the graft union, or the differential absorption ability of rootstock for nutrients (Liu et al., 2009).

## Conclusion

Based on metabolomic analysis, we identified 20 carbohydrates, 26 organic acids and 20 amino acids. Due to the effect of grafting, 35, 23, 20, 33 metabolites including sugars, amino acids and organic acids significantly changed at 10, 18, 26 and 34 DAP respectively. In addition, we identified 21 DEGs related to glucose and sucrose (*FBA2, FK, SuSy, SPS, IAI, AI, SWT3b*), malic acid (*ALMT13, ALMT8*), and amino acids metabolism (*NAOD, GS, AGT, TaT, aDH1, OGDH, aDC, 4CL 1, PaL, CaT*). These DEGS can play an important role in mediating the fruit quality by regulating sugar and acid contents in grafted watermelon. This study provides a foundation for the understanding the molecular mechanism involved in grafted watermelon influencing fruit quality and may play an important role in marker-assisted breeding of high-quality rootstock. Future studies should focus on the identification and transmission of signaling factors across the graft union, and their role in gene regulation.

## Supplemental Information

10.7717/peerj.8259/supp-1 Supplemental Information 1 The fruits of pumpkin grafted watermelon (grafted watermelon) and ungrafted watermelon at four developmental stages including 10, 18, 26, 34 Days after pollination (DAP).

10.7717/peerj.8259/supp-2 Supplemental Information 2 Hierarchical clustering analysis of DEGs between different developmental stages of ungrafted and grafted. N1-VS-N2, N1-VS-N3, N1-VS-N4, P1-VS-P2, P1 -VS-P3, P1 -VS-P4, ’a’ was the control and ’b’ was experimental group in a-VS-b. Each line refers to da.

10.7717/peerj.8259/supp-3 Supplemental Information 3 Comparison of pH between grafted and ungrafted watermelon during course of fruit development.

10.7717/peerj.8259/supp-4 Supplemental Information 4 Comparison of pH between ungrafted and pumpkin grafted watermelon during the course of fruit development.

10.7717/peerj.8259/supp-5 Supplemental Information 5 Comparison of soluble solids contents between ungrafted and pumpkin grafted watermelon during the course of fruit development.

10.7717/peerj.8259/supp-6 Supplemental Information 6 Comparison of amino acids contents between ungrafted and pumpkin grafted watermelon during course of fruit development. Green color represents abundant amino acids. Red represents total amino acid contents. DAP (Days after pollination).

10.7717/peerj.8259/supp-7 Supplemental Information 7 Comparison of sugars contents between ungrafted and pumpkin grafted watermelon during the course of fruit development. Green represents abundant sugars. Red represents total sugar contents. DAP (Days after pollination).

10.7717/peerj.8259/supp-8 Supplemental Information 8 Comparison of organic acids contents between ungrafted and pumpkin grafted watermelon during the course of fruit development. Green represents abundant organic acids. Red represents total organic acids content. DAP (Days after pollination).

10.7717/peerj.8259/supp-9 Supplemental Information 9 Principle Component Analysis (PCA) loadings and score plot data for metabolites between ungrafted and pumpkin grafted watermelon during the course of fruit development.

10.7717/peerj.8259/supp-10 Supplemental Information 10 DEGs in all sample comparison.

10.7717/peerj.8259/supp-11 Supplemental Information 11 Go Classification of all sample comparisons.

10.7717/peerj.8259/supp-12 Supplemental Information 12 The KEGG Pathways represented by all the DEGs (Differentially expressed genes).

10.7717/peerj.8259/supp-13 Supplemental Information 13 List of DEGs involved in sugar, organic acid, amino acid metabolisms and transport.

10.7717/peerj.8259/supp-14 Supplemental Information 14 List of differentially expressed transporters.

10.7717/peerj.8259/supp-15 Supplemental Information 15 Relative Expression from qRT-PCR.

## Additional Information and Declarations

### Data Availability

The following information was supplied regarding data availability: The raw data is available at GenBank: PRJNA543725.
