# Supplementary figures and images for: Comparative analysis of primary metabolites and transcriptome changes between ungrafted and pumpkin-grafted watermelon during fruit development
Source: PeerJ. 2020 Jan 6;8:e8259. doi: 10.7717/peerj.8259 (PMC6951286; doi:10.7717/peerj.8259)

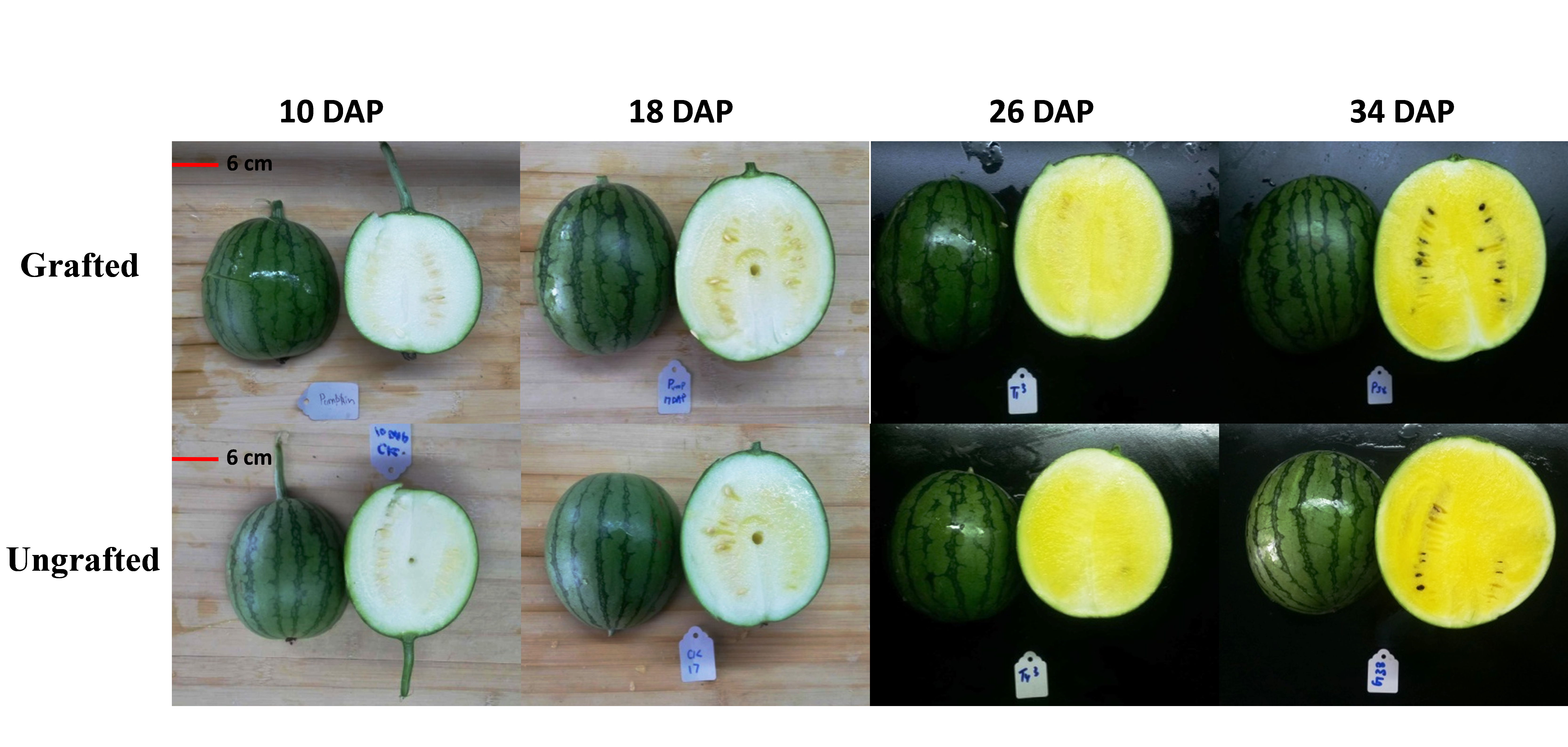

Supplement: Supplemental Information 1 [file peerj-08-8259-s001.png]

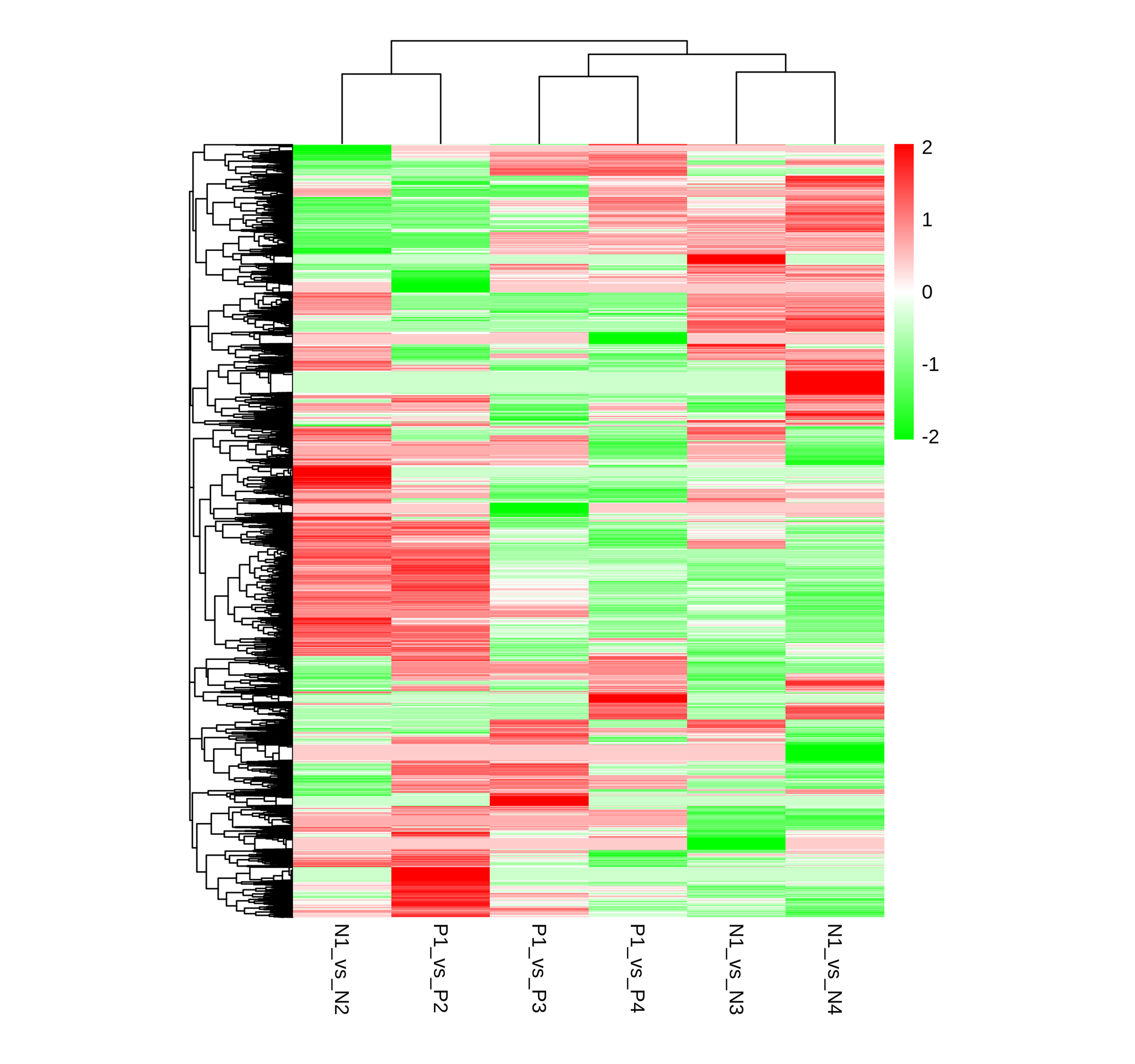

Supplement: Supplemental Information 2 [file peerj-08-8259-s002.png]
